# How Histopathology Can Contribute to an Understanding of Defense Mechanisms against Cryptococci

**DOI:** 10.1155/2013/465319

**Published:** 2013-08-22

**Authors:** Yoichiro Okubo, Naobumi Tochigi, Megumi Wakayama, Minoru Shinozaki, Haruo Nakayama, Takao Ishiwatari, Kayoko Shimodaira, Tetsuo Nemoto, Hideaki Ohno, Yukihiro Kaneko, Koichi Makimura, Katsuhisa Uchida, Yoshitsugu Miyazaki, Hideyo Yamaguchi, Kazutoshi Shibuya

**Affiliations:** ^1^Department of Surgical Pathology, Toho University School of Medicine, 6-11-1 Omori-Nishi, Ota-Ku, Tokyo 143-8541, Japan; ^2^Department of Neurosurgery, Toho University Ohashi Medical Center, 2-17-6 Ohashi, Meguro, Tokyo 153-8515, Japan; ^3^Department of Chemotherapy and Mycoses, National Institute of Infectious Diseases, 1-23-1 Toyama, Shinjuku-Ku, Tokyo 162-8640, Japan; ^4^Laboratory of Space and Environmental Medicine, Graduate School of Medicine, Teikyo University, 2-11-1 Kaga, Itabashi, Tokyo 173-8605, Japan; ^5^Teikyo University Institute of Medical Mycology, Hachioji, Tokyo 192-0395, Japan; ^6^Department of Dermatology, Peking University First Hospital, Beijing, China

## Abstract

Invasive fungal infections, particularly those considered opportunistic, have become a common and significant complication of procedures performed in advanced contemporary medicine. Among such infections, cryptococcosis, which is usually caused by infection with *Cryptococcus neoformans* and *Cryptococcus gattii*, is particularly problematic because this fungal infection occurs in immunocompromised and apparently immunocompetent individuals. It has been largely accepted that *Cryptococcus* species are recognized by cellular receptors and that Th1-type immune responses play an important role in defense mechanisms against the yeast. However, the interaction between the yeast and host tissue varies depending on the characteristics of the yeast and the immune status of the host. To gain a better understanding of the pathophysiology of cryptococcosis, we wish to emphasize the usefulness of histopathological examinations, because it allowed more detailed information of an extremely complex interaction between the causative yeasts and tissue response. In the present review, we describe the pathophysiology of cryptococcosis as largely revealed in our previous histopathological investigations of the experimental infection.

## 1. Introduction

The increasing use of invasive monitoring and aggressive therapeutic technologies in developed countries has resulted in improved survival of individuals with life-threatening diseases [1–3]. However, it has also resulted in an increased number of patients at risk of invasive fungal infections, including cryptococcosis. Cryptococcosis is a localized or systemic fungal infection mainly caused by *Cryptococcus neoformans* (*C. neoformans*) and *Cryptococcus gattii* (*C. gattii*). *C. neoformans* is widely distributed throughout the world [4], whereas *C. gattii* has been limited in tropical and subtropical regions [5]. However, there was an outbreak of *C. gattii* infection in a temperate region of British Columbia in 1999, which expanded towards US Pacific Northwest and Japan [5–10].

Recent molecular biological investigations have contributed significantly toward an elucidation of the pathogenesis of the yeast. It has been largely accepted that *Cryptococcus* species are recognized by cellular receptors and that Th1-type immune responses play an important role in defense mechanisms against the yeast [11]. However, this defense mechanism may vary among individuals, depending on the agents, tissues involved, and immune status of the host [12, 13]. Therefore, experimental animal models remain essential for an elucidation of the pathophysiology of cryptococcosis. In particular, histopathological examination of lesions at the site of infection and altered structures generally reveals an extremely complex interaction between the causative microbes and tissue response [14]. In the present review, we describe the pathophysiology of cryptococcosis as largely revealed in our previous histopathological investigations using an experimental animal model of cryptococcosis.

## 2. Elucidation of Virulence Factors of *Cryptococcus neoformans*

Primary foci of a cryptococcal infection tend to be established in the lungs before disseminating to the central nervous system [15]. To determine the pathogenesis of pulmonary cryptococcosis, we conducted histopathological investigations using mouse models that had been infected with either *C. neoformans* TIMM 0362, a relatively high-virulence and thus high-mortality strain, or TIMM 0372, a relatively low-virulence and thus low-mortality strain, via the intranasal route of infection [16]. 

**Figure 1:**
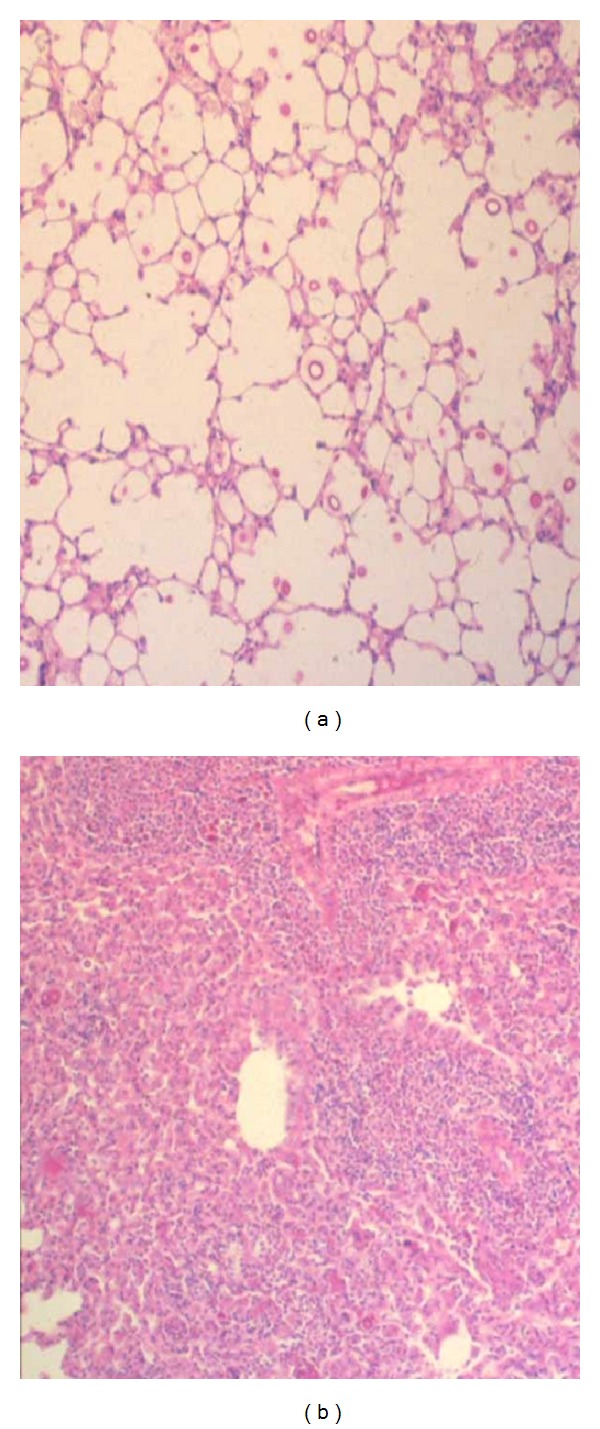
(a) The lung of mice infected with *C. neoformans* TIMM 0362 showed yeast cell proliferation in the alveoli but showed a very limited response of macrophage (13 days after infection, hematoxylin and eosin (HE) double stain, and original magnification ×200). (b) The lung of mice infected with *C. neoformans* TIMM 0372 showed numerous multinucleated giant cells (MGCs) due to the prominent macrophage response (13 days after infection, HE double stain, and original magnification ×200).

**Figure 2:**
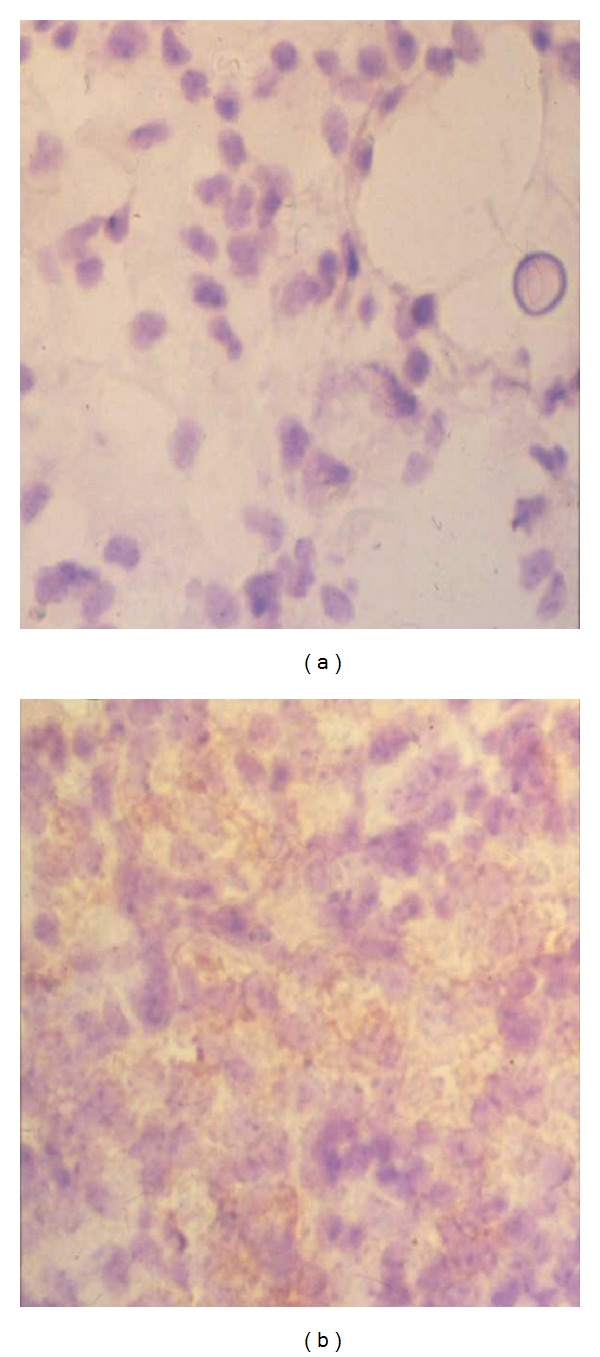
(a) Macrophages in the lung of mice infected *C. neoformans* TIMM 0362 showed no expression of MHC class II IAd molecule (immunohistochemistry, original magnification ×400). (b) Macrophages in the lung of mice infected with *C. neoformans* TIMM 0372 expressed the molecule MHC class II IAd molecule (immunohistochemistry, original magnification ×400).

**Figure 3:**
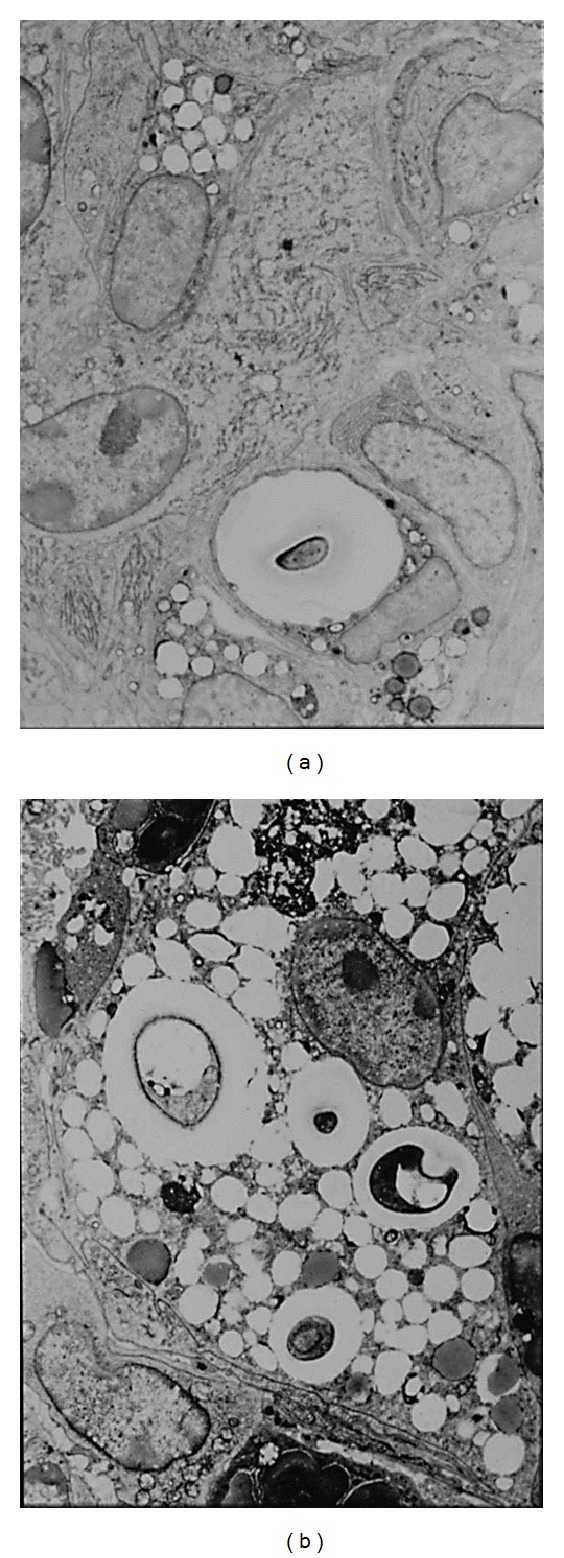
(a) An ultrastructural examination of the macrophage in mice infected with *C. neoformans* TIMM 0362 showed that a large number of phagocytized lipid droplets and a small number of the yeasts, as well as lesser developed organelles. (b) An ultrastructural examination of the macrophage in mice infected with *C. neoformans* TIMM 0372 showed a large number of developed organelles, including rough endoplasmic reticulum and Golgi.

**Figure 4:**
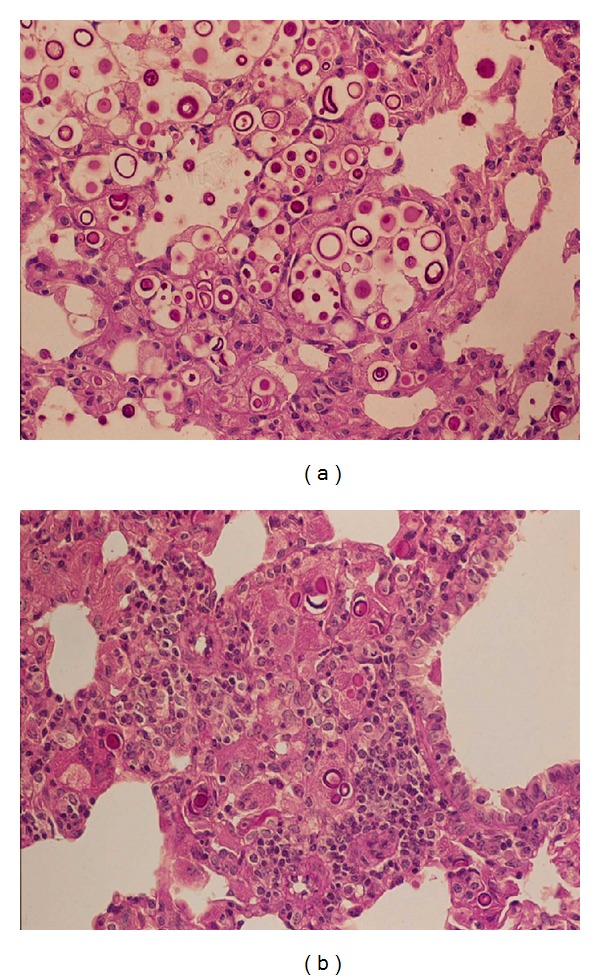
(a) Numerous and irregular-sized yeasts were diffusely observed in the pulmonary sections of cryptococcosis in homozygous (nu/nu) nude mice, but no multinucleated giant cell (MGC) was observed (periodic acid Schiff reaction, original magnification ×200). (b) MGCs with a well-developed and dense eosinophilic cytoplasm containing the yeasts were observed in the pulmonary sections of cryptococcosis in heterozygous (nu/−) nude mice (periodic acid Schiff reaction, original magnification ×200).

**Figure 5:**
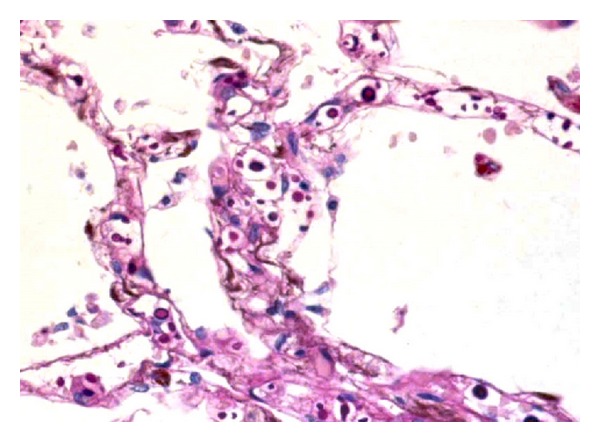
In patient with acquired immunodeficiency syndrome, a large number of yeast were observed in the pulmonary septal capillaries (periodic acid Schiff reaction, original magnification ×400).

Histopathological examination of the lungs of mice infected with *C. neoformans* TIMM 0362 showed yeast cell proliferation in the alveoli and a lesser macrophage response (Figure 1(a)). Time-dependent histopathological examination revealed decreased macrophage response but increased yeast-cell proliferation and alveolar space expansion with disease progression. Ultimately, fatal infection was established that involved not only the lungs but also the brain and several other organs. In contrast, the lungs of mice infected with *C. neoformans* TIMM 0372 showed numerous yeast-containing multinucleated giant cells (MGCs) due to the prominent macrophage response (Figure 1(b)). Time-dependent histopathological examination revealed a decrease in the number of yeast cells and in the macrophage response at the late phase of infection, but little or no alveolar expansion throughout the observation period. The most significant histopathological findings in the lungs of mice infected with *C*. *neoformans* TIMM 0362 were poor macrophage response against invasion of yeast cells, subsequent structural alteration of the lungs, and induction of cerebral cystic lesions by fatal disseminated infection. To elucidate the poor macrophage response following infection by* C. neoformans* TIMM 0362, we focused on the cell-mediated immune response against the yeast. The interaction of major histocompatibility complex (MHC) class II molecules on the surface of the antigen-presenting cells (APCs) and CD4-positive T lymphocytes plays a central role in regulating the cell-mediated immune response against infection with the *Cryptococcus* species [11, 17]. We therefore investigated the expression of MHC class II IAd molecule on the macrophage in the lungs of mice infected with *C. neoformans* TIMM 0362 and TIMM 0372 by immunohistochemical examination. As a result, the former showed no expression of MHC class II IAd molecule, and the later expressed this molecule (Figure 2). In addition, an ultrastructural examination of the macrophage in the lung of mice infected with *C. neoformans* TIMM 0362 showed a small number of phagocytized yeasts and well-preserved organelles, including rough endoplasmic reticulum and Golgi (Figure 3(a)). In contrast, that with *C. neoformans* TIMM 0372 showed a large number of phagocytized yeasts, lipid droplets, and vacuolization (Figure 3(b)). These findings suggested that poor macrophage response confirmed by histopathological examinations implies the impaired Th1 dominant cell-to-cell interaction triggered by the recognition of APCs. In fact, similar phenomenon is confirmed in the nude mice. Namely, pulmonary cryptococcosis in homozygous (nu/nu) nude mice could not induce macrophage response (Figure 4(a)), whereas that in heterozygous (nu/−) nude mice could induce macrophage response with MGC formation (Figure 4(b)). Furthermore, similar histopathological findings were also observed in the lung of patients with acquired immunodeficiency syndrome (AIDS) [12, 18]. It has been well known that development of AIDS following infection with the human immunodeficiency virus (HIV) is associated with impaired cell-mediated immune response due to a reduction in CD4-positive T lymphocytes [19–21]. Histopathological examinations indicated that the absence of CD4-positive T lymphocytes the decreasing function of antigen-presenting activity in macrophages of patients with AIDS [12], and a large number of yeasts in the septal capillaries were observed (Figure 5). 

The overall findings indicate that a Th1 dominant cell-to-cell interaction triggered by the macrophage recognition is one of the most important defense mechanisms against cryptococci. Our previous investigation revealed that inhaled *C. neoformans* TIMM 0362 can easily proliferate in alveoli due to the much lesser recognition by macrophages or other APCs via the MHC class II molecule, thereby causing greater structural alteration of the lungs and, ultimately, development of disseminated cryptococcosis. Consequently, poor cryptococcal yeast-cell recognition via macrophage or other APCs may be closely associated with the virulence of *C. neoformans*.

## 3. Differences in Pathophysiology between *C. neoformans* and *C. gattii *Infection

*C. gattii*, which was responsible for the cryptococcosis outbreak on Vancouver Island, Canada, causes life-threatening infections in immunocompetent individuals, whereas *C. neoformans* typically causes fatal diseases only in immunocompromised individuals [22]. To elucidate the differences between* C. gattii* and* C. neoformans* infection, we previously investigated and compared the biological characteristics of the pathogens and the pathophysiology of mice infected with *C. neoformans* H99, *C. gattii* 5815, and *C. gattii* R265 [23] which is causing an outbreak on Vancouver Island, Canada [6]. The mortality rate in mice infected with *C. neoformans* H99 and *C. gattii* R265 was significantly higher than that of mice infected with *C. gattii* 5815. However, there was no significant difference in mortality rate between mice infected with *C. gattii* R265 and *C. neoformans* H99. We examined certain biological characteristics. The results showed that only the growth rate under physiological conditions suggested a correlation with the mortality rate of infected mice. 

**Figure 6:**
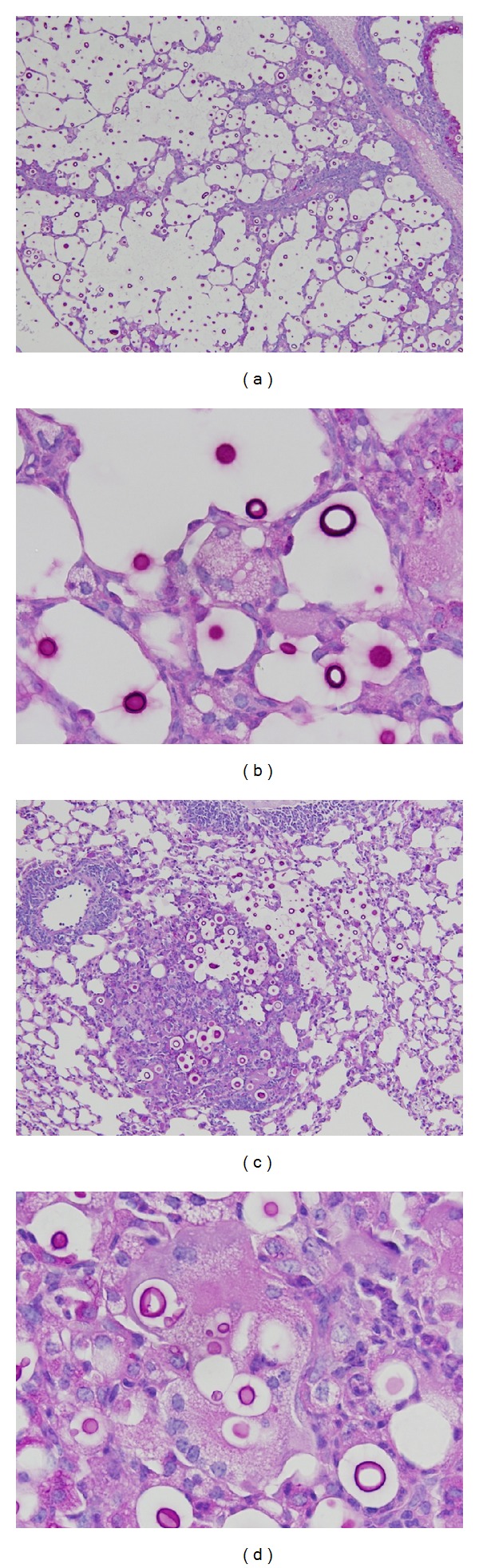
(a) Pulmonary sections of mice infected with *C. gattii* R265 exhibited eccentric pulmonary enlargement (periodic acid Schiff (PAS) reaction, ×200). (b) Pulmonary sections of mice infected with *C. gattii* R265 showed multinucleated giant cells with a foamy cytoplasm, and a small number of nuclei that were loosely aggregated (PAS reaction, ×1000). (c) Pulmonary sections of mice infected with *C. neoformans* H99 showed multiple well-demarcated nodular lesions, but no alveolar expansion was observed (PAS reaction, ×200). (d) Pulmonary sections of mice infected with *C. neoformans* H99 showed multinucleated giant cells with a well-developed and dense eosinophilic cytoplasm and a large number of nuclei (PAS reaction, ×1000).

We also conducted a detailed histopathological examination. We found a significant difference in histopathological findings of the lung between mice infected with *C. gattii* R265 and *C. neoformans* H99. In particular, the former infection showed a very limited macrophage response, but eccentric enlargement of the alveolar space was prominent due to extensive proliferation of the yeast (Figures 6(a) and 6(b)), while the latter infection showed an extensive macrophage response with giant cell formation (Figures 6(c) and 6(d)). These results may suggest that extensive proliferation of yeast in alveoli and subsequent alveolar expansion during *C. gattii* R265 infection and extensive macrophage response to intruding* C. neoformans* H99 result in a significant decrease of the pulmonary gas-exchange function of alveolar septa. This highlights the fact that the pathophysiology leading to death in *C. gattii* infection differs significantly from that of *C. neoformans* infection, whereas mice infected with both strains exhibited the same mortality rate.

We now wish to describe the difference between the lungs of mice infected with *C. gattii* and *C. neoformans*. We found that sections of lungs from mice infected with both strains of *C. gattii* showed alveolar expansion, and the periphery of enlarged alveoli was usually encompassed by collapsed septa lying on top of each other. In contrast, sections of lungs from mice infected with *C. neoformans* H99 showed no alveolar expansion. This unique difference was also confirmed by our cross-point interval analysis, which is regarded as an indicator of structural alteration of the lung [24]. The results suggest that alveoli intruded by inhaled cryptococci were expanded due to extensive yeast proliferation, and that *C. gattii* can cause a greater structural alteration of the lung than *C. neoformans*.

Our study revealed that the poorest macrophage response was found in mice infected with *C. gattii* R265. It suggest that *C. gattii* R265 may have the poorest potential to induce a recognition cascade in the immune system which is consistent with a previous report [25]. We therefore conducted morphometric analyses of MGCs to quantitatively determine the strength of the macrophage response. We found that the findings of histopathological examinations may imply that *C. gattii* possesses some unknown mechanisms that allow it to avoid or escape recognition by macrophages or other antigen-presenting cells.

**Figure 7:**
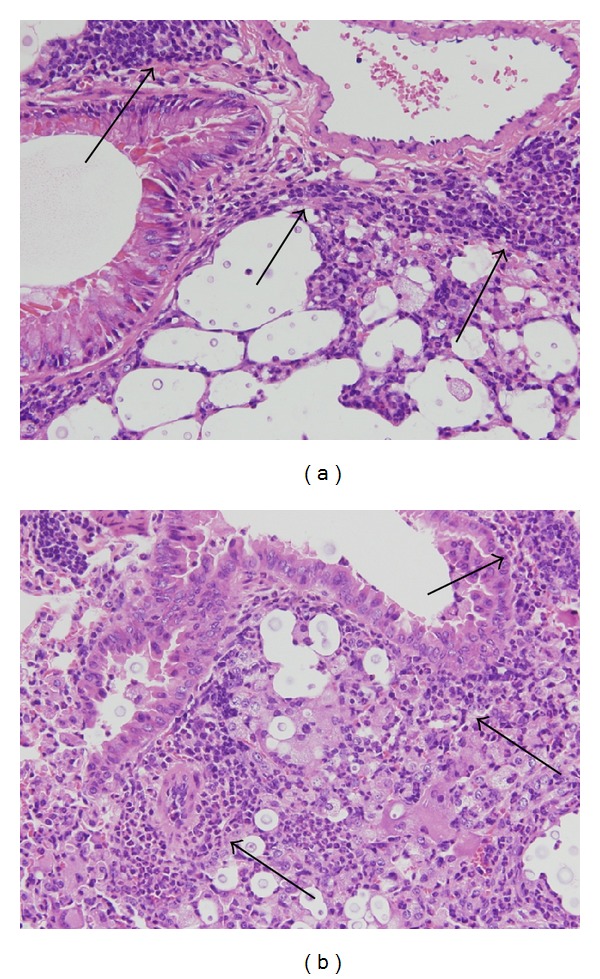
(a) Pulmonary sections of mice infected with *C. gattii* R265 exhibited poor neutrophil recruitment around the bronchovascular structures (arrowheads, hematoxylin and eosin (HE) double stain, and ×400). (b) Pulmonary sections of mice infected with *C. neoformans* H99 showed extensive neutrophil recruitment around the bronchovascular structures (arrowheads, HE double stain, and ×400).

We now wish to provide more detailed discussion on the pathophysiology of cryptococcosis by comparing the previous investigation. Cheng et al. reported the histopathological differences between the mice infected with *C. gattii *R265 and *C. neoformans* H99 [26]. In their report, no significant difference was found for mortality rate between mice infected with *C. gattii* R265 and *C. neoformans* H99, as well as our present study. In addition, they suggested that *C. neoformans* H99 induces extensive neutrophil recruitment around the bronchovascular structures, but the ability was poor in *C. gattii *R265. Certainly, the same finding was also confirmed in our experimental murine model of cryptococcosis (Figures 7(a) and 7(b)). Since it has been reported that the migration of neutrophils into lung tissue is important in the early protection of mice against progressive cryptococcosis [27], the degree of neutrophil recruitment may be related to difference of pathophysiology between *C. gattii *and *C. neoformans* infections.

On the other hand, Cheng et al. reported that no significant difference was found in number of macrophages between *C. gattii* R265 and *C. neoformans* H99 infected mice, whereas our study revealed that macrophage response of the mice infected with *C. gattii* R265 was poor, but that infected with *C. neoformans* H99 was extensive. The contradictory result may attribute to the difference of the experimental design and analysis methods. There were two important differences: one is routes of pulmonary infection (intranasal infection versus intratracheal infection), and another is term of infection (7 days versus 14 days). Since we were able to observe histopathological findings of the lesion just before death of the infected mice, macrophage response could be amplified in our study. In addition, Cheng et al. employed flow-cytometric analysis to count the number of macrophages that is superior to manual counting of the cell, but it cannot count the number of macrophages within MGC, accurately. Since macrophage fused together to form a MGC, the flow-cytometric analysis was unable to accurately assess the macrophage response.

Consequently, there is an extensively different pathophysiology leading to death between *C. gattii* and *C. neoformans* infections. Namely, both extensive proliferation of the yeast in alveoli during *C. gattii* R265 infection and macrophage response to intruding* C. neoformans* H99 would cause respiratory failure of the mice. Despite the same mortality rates between mice infected with *C. gattii* R265 and *C. neoformans* H99 was found, the pathophysiology leading to death between *C. gattii *and* C. neoformans* infection was different, which was only revealed by histopathological examination. We therefore wish to emphasize the usefulness of the yield of the histopathological examination for experimental animal model.

## 4. Exploration to Know Specific Virulence Factors of *C. gattii*

We found that *C. gattii* is characterized by its ability to induce high pulmonary structural alteration [23]. To gain an understanding of the cause of this unique feature, we investigated the characteristics of seven strains of *C. gattii* (TIMM 4097 and TIMM 4901 to 4906) [28, 29].* C. gattii* TIMM 4097, TIMM 4901 to 4906 were isolated from Japanese zoo-bred koalas. Serotypes of the strain were confirmed using slide agglutination tests according to the manufacturer's instructions (Cryptocheck Iatron RM 304-K kit; Mitsubishi Kagaku Iatron, Inc., Tokyo, Japan). All animal studies were performed in accordance with the guidelines and permission of the animal experiment care committee of the Teikyo University. On day 100 after infection, the cumulative mortality rates in mice infected with *C. gattii* TIMM 4097 was 10/10 (100.0%), whereas the rates in mice infected with *C. gattii* TIMM 4901 to 4906 were 0/10 (0.0%). According to Kaplan-Meier survival analysis with a log-rank significance test, the mortality rate in mice infected with *C. gattii* TIMM 4097 was significantly higher than that of mice infected with *C. gattii* TIMM 4901 to 4906. 

To elucidate factors affecting virulence of the yeast, we examined biological characteristics of seven strains of *C. gattii*, including growth rate, capsule thickness, melanin production, and hydrolase (urease, proteinase, and phospholipase) activity. Despite the significant differences in mortality rate among mice infected with the different strains, no significant differences were found among the strains regarding any of the characteristics examined, except for growth rate. The results showed that only the growth rate suggested a correlation with the mortality rate of infected mice.

Since previous investigations have reported that the molecular type of a *C. gattii* strain plays a strong role in its virulence [22], we investigated the molecular type of seven strains of *C. gattii *using multilocus sequence typing (MLST) analysis in accordance with the consensus *C. gattii* typing scheme established by the Cryptococcal Working Group I of the International Society for Human and Animal Mycology (ISHAM) [30], which includes the following seven unlinked genetic loci: *CAP59, GPD1, LAC1, PLB1, SOD1, URA5,* and *IGS1*. Although the mortality rate of mice infected with *C. gattii* TIMM 4907 was significantly higher than that of mice infected with *C. gattii* TIMM 4901 to 4906, MLST analysis revealed that the molecular type of all seven strains was VG I (Table 1), indicating that the virulence of these *C. gattii* strains may not be regulated by their molecular type.

**Figure 8:**
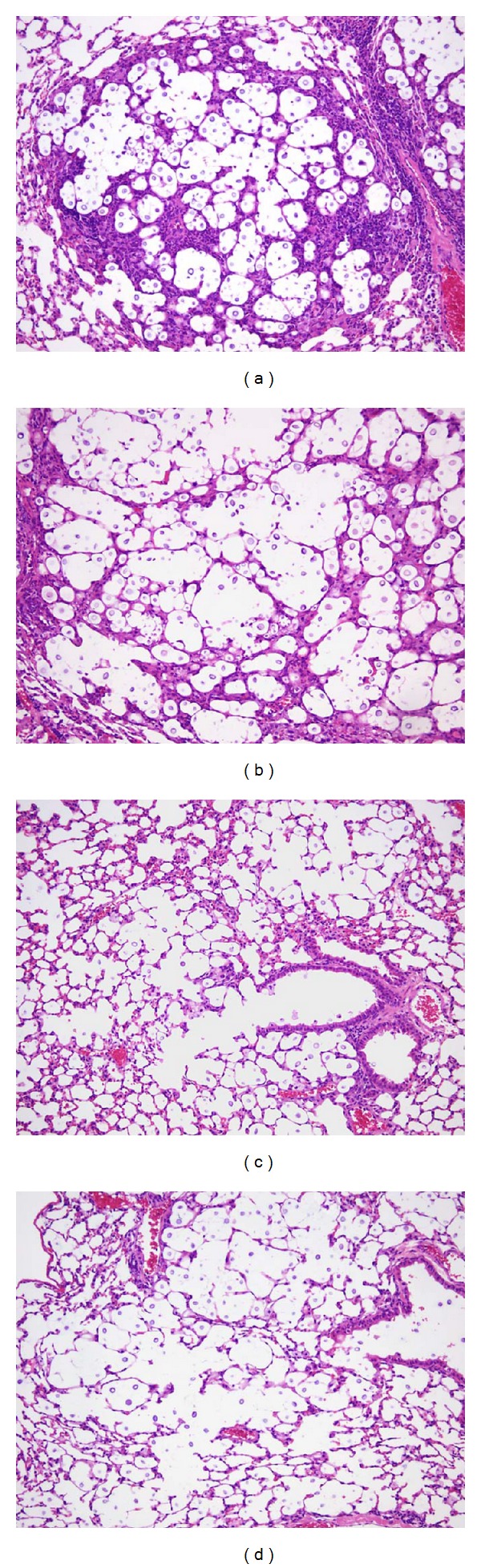
(a, b) On days 15 and 30 after infection, pulmonary sections of mice infected with *C. gattii* TIMM 4097 showed eccentric enlargement of alveoli containing prominent proliferation of yeast cells with disease progression (periodic acid Schiff (PAS) reaction, ×100, resp.). (c, d) On days 15 and 30 after infection, pulmonary sections of mice infected with *C. gattii* TIMM 4903 showed lesser degree of yeast cell proliferation and alveolar expansion than these with *C. gattii* TIMM 4097 (PAS reaction, ×100, resp.).

Whereas there were no differences in biological characteristics among the seven *C. gattii *strains of VG I isolated from Japanese zoo-bred koalas, the mortality rate of mice infected with *C. gattii* TIMM 4097 was significantly higher than that of mice infected with other six *C. gattii* strains. Therefore, we conducted detailed histopathological examination to gain an insight into the pathophysiology underlying the virulence. As the *C. gattii* TIMM 4901 to 4906 strains were found to have the same phenotypic expression, molecular type, and mortality rate in infected mice, *C. gattii* TIMM 4903 was randomly selected as a representative type for further examination and comparison with *C. gattii* TIMM 4097. Time-dependent histopathological examination revealed that pulmonary sections obtained from mice infected with *C. gattii* TIMM 4097 showed eccentric enlargement of alveoli containing prominent proliferation of yeast cells with disease progression (Figures 8(a) and 8(b)). In contrast, pulmonary sections of mice infected with *C. gattii* TIMM 4903 showed little or no alveolar expansion and contained a smaller number of yeast cells than sections of mice infected with *C. gattii* TIMM 4097 (Figures 8(c) and 8(d)), and quite interestingly, several yeast cells in the bronchi. 

**Figure 9:**
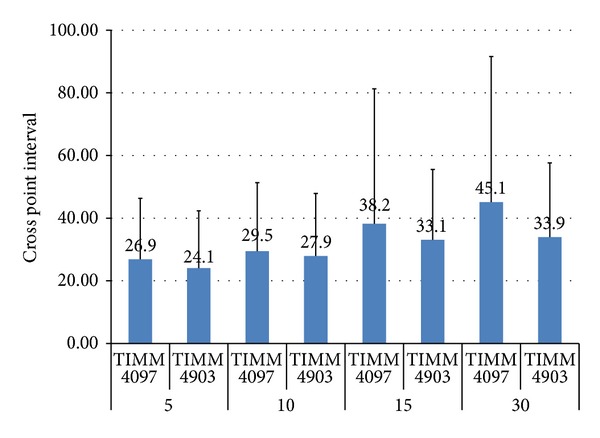
The mean ± standard  deviation (SD) values of the cross-point interval of the lungs of the mice infected with *C. gattii *TIMM 4097 on days 5, 10, 15, and 30 after infection were 26.86 ± 19.48, 29.46 ± 21.89, 38.21 ± 43.08, and 45.13 ± 46.48 *μ*m, respectively. In contrast, that of mice infected with *C. gattii *TIMM 4097 were 24.06 ± 18.30, 27.90 ± 20.00, 33.07 ± 22.49, and 33.92 ± 23.70 *μ*m, respectively. Analyses of the mean value and variance of the cross-point interval revealed significant differences between the mean value and variance of mice infected with *C. gattii* TIMM 4097 and 4903 on days 15 and 30 after infection.

To gain a detailed understanding of the structural alteration of the lungs, the cross-point interval was measured. Analyses of the mean value and variance of the cross-point interval revealed significant differences between the mean value and variance of mice infected with *C. gattii* TIMM 4097 and 4903 on days 15 and 30 after infection (Figure 9). These results suggest that although alveoli invaded by inhaled cryptococci of any strain expand due to extensive yeast proliferation, inhalation of *C. gattii* TIMM 4097 may cause greater structural alteration of the lungs than *C. gattii* TIMM 4903 at a late phase of infection, which may be due to the greater ability of *C. gattii* TIMM 4097 to reside in the alveolar lumen compared to *C. gattii* TIMM 4903.

**Figure 10:**
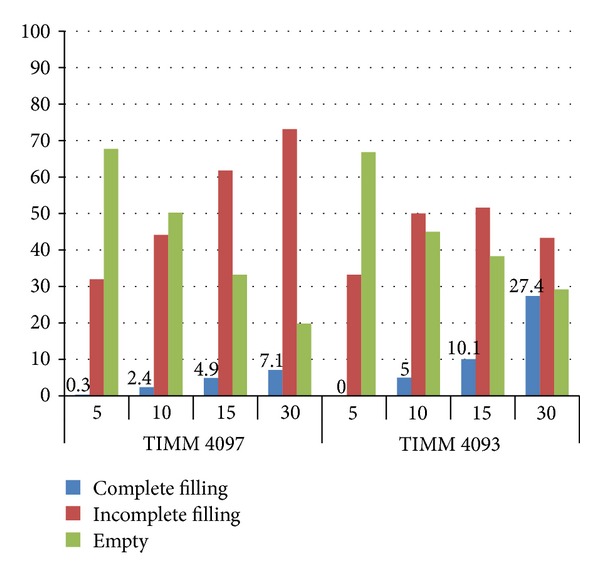
The percentage of bronchi that was “completely full” in mice infected with *C. gattii* TIMM 4097 on days 10, 15, and 30 after infection was 2.4, 4.9, and 7.1%, respectively, while that of mice infected with *C. gattii* 4903 was 5.0, 10.1, and 27.4%, respectively. Results revealed that the ratio of “completely full” bronchi in mice infected with *C. gattii* TIMM 4903 was significantly higher than that of mice infected with *C. gattii *TIMM 4097 throughout the observation period.

**Figure 11:**
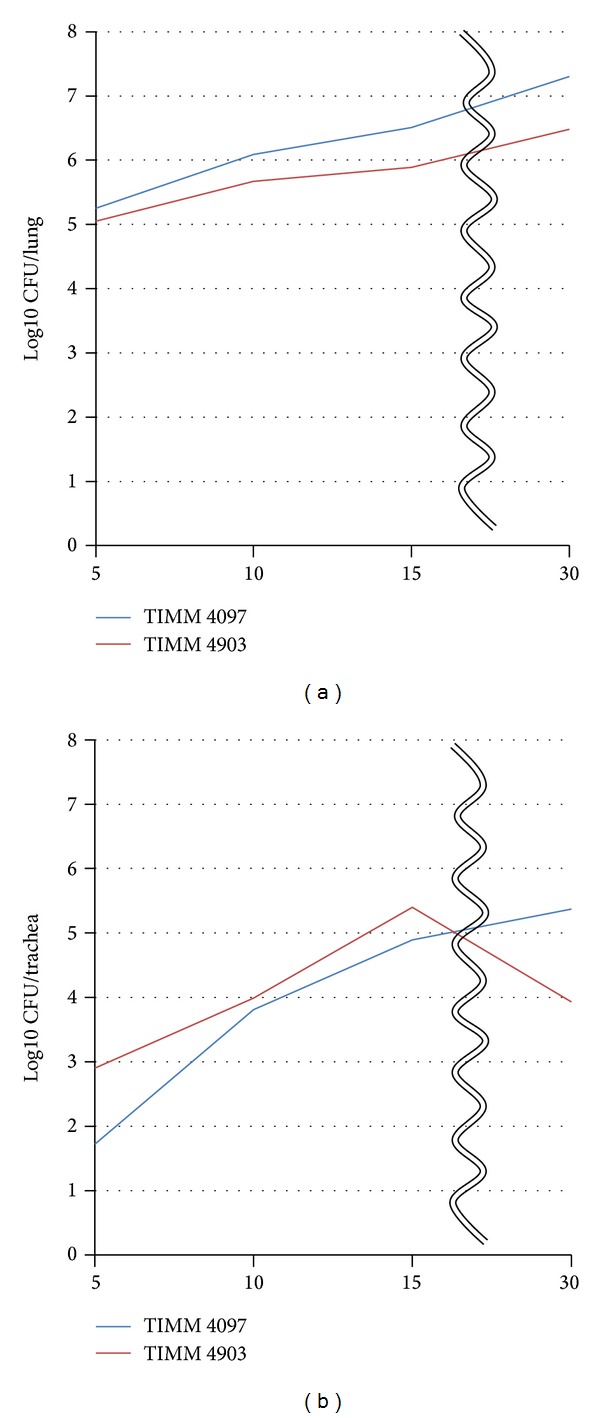
The mean ± SD log_10_ CFU/mL counts in the lungs of mice infected with *C. gattii* TIMM 4097 on days 5, 10, 15, and 30 after infection were 5.25 ± 0.16, 6.09 ± 0.05, 6.51 ± 0.12, and 7.30 ± 0.08, respectively, similar to those of mice infected with *C. gattii* TIMM 4903, which were 5.05 ± 0.19, 5.67 ± 0.14, 5.89 ± 0.12, and 6.48 ± 0.24, respectively. In contrast, the mean ± SD log_10_ CFU/mL counts in the tracheae of mice ys 5, 10, 15, and 30 after infection were 1.72 ± 0.54, 3.81 ± 0.11, 4.89 ± 0.60, and 5.37 ± 0.18, respectively, similar to those of mice infected with *C. gattii* TIMM 4903, which were 2.90 ± 0.14, 3.99 ± 0.04, 5.40 ± 0.10, and 3.93 ± 0.52, respectively. Statistical analysis revealed that the mean log_10_ CFU count in the lungs of mice infected with *C. gattii* TIMM 4097 was significantly higher than that of mice infected with *C. gattii *TIMM 4903 in each evaluation period except for day 5 after infection. Nevertheless, the mean log_10_ CFU counts in the tracheae of mice infected with *C. gattii* TIMM 4903 on days 5, 10, and 15 after infection were significantly higher than those of mice infected with *C. gattii* 4097.

The results of the histopathological examination and morphometric analysis of the lungs of infected mice indicated that infection with *C. gattii* TIMM 4097 (a high-virulence strain) tend to induce greater alteration of lung structure than infection with *C. gattii* TIMM 4903 (a low-virulence strain). To investigate the hypothesis that *C. gattii* TIMM 4097 tends to reside in the alveoli, the extent to which the yeast cells filled the bronchi was determined by semiquantitative assessment. All bronchi of the pulmonary PAS-stained sections were observed, and the bronchi were classified as “empty,” “partially full,” or “completely full” based on the degree to which the bronchi were filled with yeast cells. Results revealed that the ratio of “completely full” bronchi in mice infected with *C. gattii* TIMM 4903 was significantly higher than that of mice infected with *C. gattii *TIMM 4097 throughout the observation period (Figure 10). This assessment of the bronchi supported our hypothesis that *C. gattii* TIMM 4097 can rigidly reside in the alveoli. To test this hypothesis, the number of viable cells in the lungs and trachea of mice infected with each strain was also counted. Statistical analysis revealed that the mean log_10_ CFU count in the lungs of mice infected with *C. gattii* TIMM 4097 was significantly higher than that of mice infected with *C. gattii *TIMM 4903 in each evaluation period except for day 5 after infection (Figure 11). Nevertheless, and quite interestingly, the mean log_10_ CFU counts in the tracheae of mice infected with *C. gattii* TIMM 4903 on days 5, 10, and 15 after infection were significantly higher than those of mice infected with *C. gattii* 4097 (Figure 11). These results support the hypothesis that *C. gattii* TIMM 4097 tends to reside in the alveoli, whereas *C. gattii* TIMM 4903 tends to be washed out from the alveoli and move into the central side of the respiratory system. 

**Figure 12:**
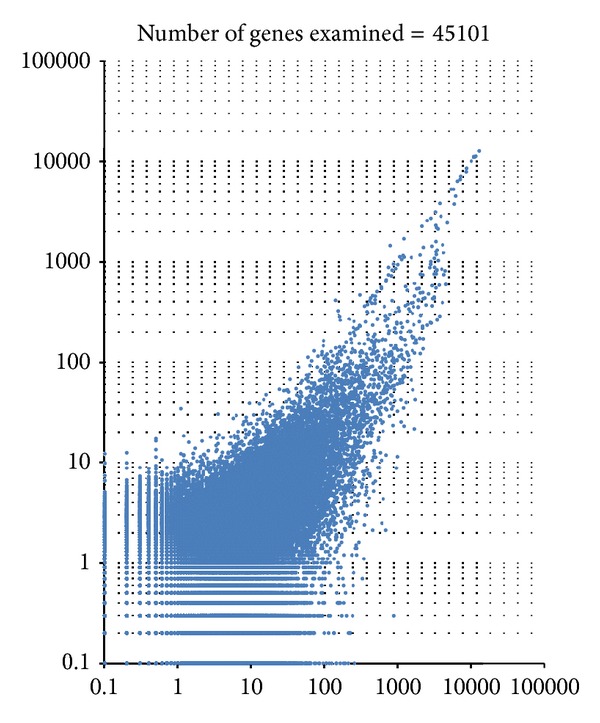
Gene expression in the lungs of mice infected with *C. gattii* TIMM 4097 and 4903 was measured and compared using microarray analysis. The microarray analysis detected the expression of 45,101 genes.

**Figure 13:**
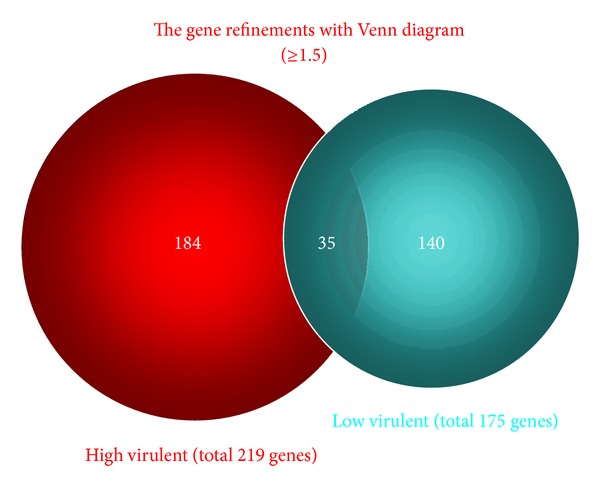
Whereas 219 genes were upregulated in the lungs of mice infected with *C. gattii* TIMM 4097 (a high-virulence strain), only 175 genes were upregulated in the lungs of mice infected with *C. gattii* TIMM 4903 (a low-virulence strain). Thus, 35 genes were upregulated in the lungs of mice infected with either *C. gattii* TIMM 4097 or 4903, while 184 genes were upregulated only in the lungs of mice infected with *C. gattii* TIMM 4097.

**Figure 14:**
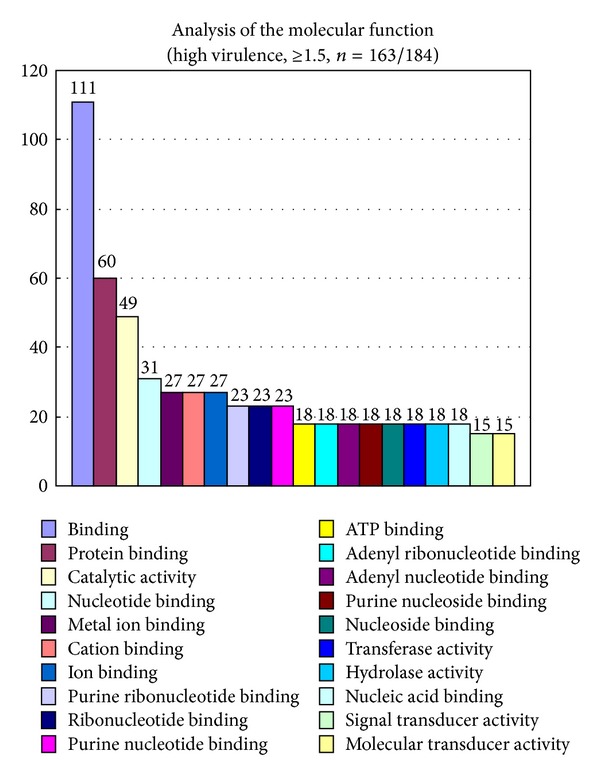
Analysis of the molecular function of stored genes was conducted using the Biological Networks Gene Ontology tool (BINGO, http://www.psb.ugent.be/cbd/papers/BiNGO/) to identify statistically overrepresented GO categories among the biological data. For the 184 genes upregulated only in the lungs of mice infected with *C. gattii* TIMM 4097, 163 terms related to molecular functioning were detected, and GO terms related to binding accounted for about 75% of all terms.

A microarray assay with gene ontology analysis was also performed with reference to the previous investigation [31]. Gene expression in the lungs of mice infected with *C. gattii* TIMM 4097 and 4903 was measured and compared using microarray analysis (Affymetrix GeneChip microarrays). All the microarray data are deposited in GEO (accession number GSE48595). The microarray analysis detected the expression of 45,101 genes (Figure 12) and the probability of the value of each data point was indicated as a “flag,” such as “present” (*P* ≤ 0 to <0.04), “marginal” (*P* ≤ 0.04 to <0.06), or “absent” (*P* ≤ 0.06 to <0.5). Gene expression that contained the “absent” flag (expression of 39,181 genes) was excluded, and the expression of 5,920 genes was extracted (Table 2). As referenced in previous investigations, a second selection was then performed using a cut-off value indicating at least a ±1.5-fold change (log 2 ratio). The results revealed that although 219 genes were upregulated in the lungs of mice infected with *C. gattii* TIMM 4097, only 175 genes were upregulated in the lungs of mice infected with C. *gattii* TIMM 4903. Thus, 35 genes were upregulated in the lungs of mice infected with either *C. gattii* TIMM 4097 or 4903, while 184 genes were upregulated only in the lungs of mice infected with *C. gattii* TIMM 4097 (Figure 13). To examine the hypothesis that upregulation of genes in mice infected with *C. gattii* TIMM 4097 is related to the high virulence of the strain, Gene Ontology (GO) analysis of the stored genes was conducted using the Biological Networks Gene Ontology tool (BINGO, http://www.psb.ugent.be/cbd/papers/BiNGO/) to identify statistically overrepresented GO categories among the biological data. For the 184 genes upregulated only in the lungs of mice infected with *C. gattii* TIMM 4097, 163 terms related to molecular functioning were detected, and GO terms related to binding accounted for about 75% of all terms (Figure 14). 

Our previous investigations suggested that *C. gattii* TIMM 4097 rigidly resides in the alveolar space and that this may cause alveolar expansion, whereas *C. gattii* TIMM 4903 is likely to be washed out from the alveoli and move into the central side of the respiratory system, as indicated by observation of little change in lung structure of mice infected with this strain. These differences may have arisen from the different capacity of the two strains to adhere onto the alveolar epithelium and is partly confirmed by our previous study.

## 5. Conclusion

The defense mechanism against cryptococci may vary among individuals, depending on the agents, tissues involved, and immune status of the host. Given these considerations, we wish to emphasize the usefulness of histopathological examinations, because it allowed more detailed information of an extremely complex interaction between the causative microbes and tissue response.

## Figures and Tables

**Table 1:** The molecular type of seven strains of *Cryptococcus gattii *was investigated using multilocus sequence typing (MLST) analysis. Although the mortality rate of mice infected with *C. gattii* TIMM 4907 was significantly higher than that of mice infected with *C. gattii* TIMM 4901 to 4906, MLST analysis revealed that the molecular type of all seven strains was VG I.

Molecular types of *Cryptococcus gattii *	
Strain	Molecular type
*C. gattii *TIMM 4097	VG I
*C. gattii *TIMM 4901	VG I
*C. gattii *TIMM 4902	VG I
*C. gattii *TIMM 4903	VG I
*C. gattii *TIMM 4904	VG I
*C. gattii *TIMM 4905	VG I
*C. gattii *TIMM 4906	VG I

**Table 2:** The probability of the value of each data point was indicated as a “flag,” such as “present” (*P* ≤ 0 to < 0.04), “marginal” (*P* ≤ 0.04 to < 0.06), or “absent” (*P* ≤ 0.06 to < 0.5). Gene expression that contained the “absent” flag (expression of 39,181 genes) was excluded, and the expression of 5,920 genes was extracted.

	Absent	Marginal	Present
Control	22562	1016	21523
*C. gattii *TIMM 4097	26505	1068	17528
*C. gattii *TIMM 4903	37445	937	6719
